# Process Parameter Selection for Production of Stainless Steel 316L Using Efficient Multi-Objective Bayesian Optimization Algorithm

**DOI:** 10.3390/ma16031050

**Published:** 2023-01-25

**Authors:** Timur Chepiga, Petr Zhilyaev, Alexander Ryabov, Alexey P. Simonov, Oleg N. Dubinin, Denis G. Firsov, Yulia O. Kuzminova, Stanislav A. Evlashin

**Affiliations:** 1 Skolkovo Institute of Science and Technology, 121205 Moscow, Russia; 2 World-Class Research Center, State Marine Technical University, 190121 Saint Petersburg, Russia

**Keywords:** metal additive manufacturing, powder bed fusion, SS316L, printing parameters, machine learning, multi-objective optimization

## Abstract

Additive manufacturing is a modern technique to produce parts with a complex geometry. However, the choice of the printing parameters is a time-consuming and costly process. In this study, the parameter optimization for the laser powder bed fusion process was investigated. Using state-of-the art multi-objective Bayesian optimization, the set of the most-promising process parameters (laser power, scanning speed, hatch distance, etc.), which would yield parts with the desired hardness and porosity, was established. The Gaussian process surrogate model was built on 57 empirical data points, and through efficient sampling in the design space, we were able to obtain three points in the Pareto front in just over six iterations. The produced parts had a hardness ranging from 224–235 HV and a porosity in the range of 0.2–0.37%. The trained model recommended using the following parameters for high-quality parts: 58 W, 257 mm/s, 45 µm, with a scan rotation angle of 131 degrees. The proposed methodology greatly reduces the number of experiments, thus saving time and resources. The candidate process parameters prescribed by the model were experimentally validated and tested.

## 1. Introduction

Metal additive manufacturing (AM) [1] is a sub-field of AM that focuses on the production of fully functional metallic parts with a complex geometry that are hard to create using conventional methods. The range of application for metal AM is from aerospace [2] to biomedical industries [3]. With recent technological advancements, it is possible to create jet engine parts [4], medical instruments [5], bone implants [6], energy storage elements [7], and more. However, there are several challenges hampering the widespread industrial adoption, one of which is the lack of the quality consistency and repeatability of the produced parts [8]. Surface quality, porosity, as well as other defects remain serious issues that can compromise the performance of the produced part [9]. An excessive amount of pores and inclusions can lead to unacceptable strength, ductility, and fatigue resistance. Ensuring the reliability and quality of metal AM products will potentially lead to high-volume production, allowing the technique to breach its niche market barrier.

Many defects and imperfections can be avoided beforehand by carefully setting the process parameters [10,11,12,13]. The key process parameters in laser-assisted powder bed fusion (L-PBF) are the laser power (*P*), scanning speed ($v_{s}$), hatch distance (*h*), and scanning strategy [14]. Together, they determine the temperature gradients, the solidification rate, and the morphology of the grains and their growth pattern, which affect the microstructure of the finished part [8]. For instance, the cooling rate of the alloy could be reduced by applying a high power *P* and low scanning speed $v_{s}$. When insufficient heat is applied, there is not enough energy to totally melt the powder particles. As a result, the particles of the solid powder tend to adhere to the build’s surfaces, resulting in “balling phenomena” [15].

One of the most commonly used metrics to compare parts manufactured with L-PBF under different conditions is the volumetric energy density (VED) [16], which is defined as:

(1) $$E=\frac{P\times \mu_{p}}{v_{s}\times h\times t}\;\left[\frac{J}{mm^{3}}\right]$$

where *P* is the laser power (W), $v_{s}$ is the scanning speed (mm/s), *h* is the hatch spacing (mm), *t* is the layer thickness (mm), and $\mu_{p}$ is the powder absorptivity, which, for this work, was set to one. It should be noted that Equation (1) has limitations. For instance, it cannot capture complex melt pool dynamics [17] and the transition from conduction to “keyhole mode” [18]. However, for the preliminary analysis and small operating windows, it serves as a good metric. Insufficient VED leads to the retention of a high number of macroscopic pores, undermining the mechanical performance of the produced part. In contrast, a high VED results in “keyhole mode”, a situation in which the melt pool is so deep, that it remelts the previous layers, which also deteriorates the fabricated part. Spattering can arise when molten droplets or powder particles are expelled from the molten pool at high power densities. Due to the local vaporization of the alloying elements, the molten pool suffers considerable recoil pressure. When this pressure is greater than the surface tension force at the liquid pool’s boundary, molten droplets may be ejected.

Selecting the process parameters by trial and error is a costly and time-consuming procedure. A more efficient way of choosing the right configuration from prior experiments involves Bayesian methods and genetic algorithms [19,20,21,22,23]. Previous studies showed the applicability of machine learning methods for the optimization in the laser powder bed fusion (L-PBF) process [24,25,26]. However, they were primarily focused on adjusting two process parameters, the laser power and scanning speed, while keeping the other parameters fixed. This study builds upon and broadens previous works, not only by considering more process parameters, but also by simultaneously optimizing several physical properties such as the hardness and porosity.

In order to meet several goals at once, multi-objective Bayesian optimization (MOBO) is often used [27,28]. One of the most-recent MOBO algorithms is diversity-guided efficient multi-objective optimization (DGEMO) [29], which performs well on benchmark problems, showing significant advantages over similar methodologies by having a good trade-off between exploration and exploitation. The working principle of DGEMO is two-fold: first, building a surrogate model of a black-box objective function based on empirically obtained data; second, an acquisition function samples points in the design space that are closest to the Pareto-optimal point. If improvements in one objective can only be made if at least one other objective value decreases, the point is said to be Pareto optimal. A hypervolume indicator [30,31], or the volume of the area of the performance space filled by the points on the Pareto front, is used to measure the improvement of the Pareto front. Determining the Pareto front with the greatest achievable hypervolume indicator is the goal of the DGEMO method. More detailed information about the underlying mathematical formulations can be found in [29].

In this work, we applied DGEMO for the process optimization of the L-PBF process using batch evaluations. The samples were printed with the parameters proposed by this method and were experimentally validated. After each iteration, the model was updated until we obtained satisfactory results.

## 2. Materials and Methods

The stainless steel powder Höganäs AB 316L was used in this experiment with the average size of the particles being 30 µm. The detailed powder properties are presented in [13].

**Figure 1:**
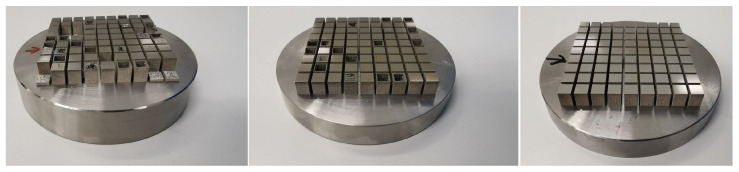
Sample of 243 specimens printed under different processing conditions.

For the purposes of the study, 636 specimens were successfully printed with a geometry of 8.0×8.0×10.0 mm${}^{3}$. The production of the specimens was carried out using the metal 3D printer Trumpf Truprint 1000 to realize the L-PBF technique. The following printing parameters were varied in the limits (see Table 1) chosen according to the printer’s technical limits and the possibility to build the solid parts. First, the parameters were changed gradually within the specified limits such that every row had only one parameter changed in small increments of ±30%. In order to improve the predictive power of the model, every single row was changed randomly for the last two platforms. The following parameters were varied: the time delay between successive layers (min), argon gas circulation speed (m/s), laser power (W), scanning speed (mm/s), hatch spacing (µm), and scan strategy, which defines the angle of rotation of the scanning path for each successive layer. The laser beam diameter and layer thickness were constant, which were 55 µm and 20 µm, respectively. The fabricated samples are shown in Figure 1. Note that, utilizing the printing parameter boundaries prescribed by the documentation sometimes resulted in poor-quality samples (as can be seen in Figure 1).

All printed samples were ground and polished for further optical porosity analysis according to ASTM E 1245. It was presented earlier that the near-surface defects were located at a depth of up to 170 µm [32]. In the present work, only the inner defects were taken into account. Therefore, at least 300 µm of the top surface layer was removed from each sample. The analysis was conducted using the optical microscope Zeiss Axio Scope.A1 and the Thixomet Pro software, which converts the images to grayscale. The ratio of the number of black to white pixels was calculated to give the final porosity estimation. The fact that optical microscopy can only assess 3D porosity as an area fraction in a specific 2D plane is a significant disadvantage. As a result, this method cannot be used to estimate the pore volume accurately. For a more precise measurement of the porosity of an entire specimen, the Archimedes method is recommended [33]. However, it takes more time and requires extra machining procedures, such as electrical discharge machining, to remove the specimens from the substrate.

The microhardness values were obtained using the Vickers microhardness testing machine ITV-1-AM (Metrotest, Russia) according to ISO 22826. The microhardness of each sample was tested at three different points under a load of 0.3 kgf.

**Figure 2:**
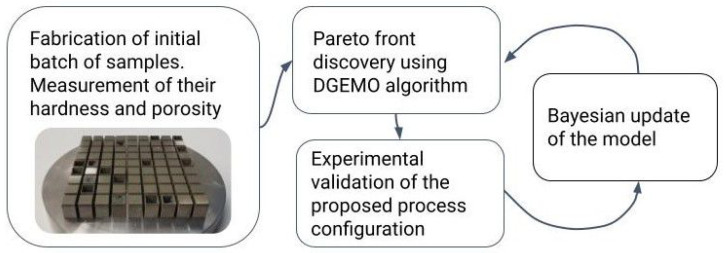
The workflow pipeline designed to find the set of optimal process parameters using DGEMO.

The mechanical properties and corresponding process configuration were recorded in a spreadsheet. Even though we initially created 636 samples, it sufficed to use a small portion of the full dataset for model training. To showcase the efficacy of the proposed method, we shuffled the 636 data entries and took the first 57 rows for model training. This subset underwent the following iterative procedure: (1) training the Gaussian-process (GP)-based surrogate model on the subset of data and mapping the relationship between the input variables (process configuration) and the target variables (hardness and porosity); (2) approximation of the Pareto front; (3) selection of the next set of candidate configurations; (4) Bayesian update of the evaluated samples, after which we returned to Step 1. This continued until we were satisfied with the results or convergence was reached. The simplified version of the working pipeline is shown in Figure 2. To speed up the research effort, the open-source automated optimal experiment design (AutoOED) was used [34]. This package includes DGEMO and other state-of-the art MOBO algorithms.

### 2.1. Gaussian Process

For each objective function (hardness and porosity), the Gaussian process regression (GPR) model was built separately. The GPR predefined a prior Gaussian distribution with mean μ and covariance *k* over the regression function *f* without a parametric form:

(2) $$f∼GP\left(\mu ,C\right)$$

The goal was to utilize the function *f* to build the relationship between the process parameters *x* and objective functions (hardness or porosity values) *Y*:

(3) $$Y=f\left(x\right)+\varepsilon$$

Using the covariance function *C* (e.g., Matern kernel function) provides a sense of similarity between the fabrication conditions as a prior. As a result, more comparable porosity and hardness values result from closer manufacturing conditions, increasing the prediction power of the GP-based surrogate model.

The training data consisted of multiple manufacturing conditions ($x_{1}$, $x_{2}$, *…*, $x_{6}$) and random variables *f* of the porosity and hardness values following a multivariate Gaussian distribution to predict the fabrication conditions and corresponding objective function values. Finally, the posterior distribution of the GP is given in the form:

(4) $$f\left(x\right)∼N\left(\mu (x),\Sigma (x)\right),$$

where the mean μ is the expected hardness or porosity and the covariance function Σ highlights the relationships between the porosity or hardness variables *f* based on the similarity between their fabrication conditions ($x_{1}$, $x_{2}$, *…*, $x_{6}$). In this work, we used the Matern kernel function [35]. The hardness or porosity as $\bar{f}$ for the new manufacturing configuration ($\bar{x_{1}},\bar{x_{2}},\ldots ,\bar{x_{6}}$) was estimated by the GPR via the joint multivariate Gaussian distribution with the random variables *f* for the manufacturing conditions in the training data.

(5) $$\left[\begin{array}{c} f\\ \bar{f} \end{array}\right]∼N\left[\begin{array}{c} \left(\begin{array}{c} \mu \\ \bar{\mu } \end{array}\right),\left(\begin{array}{cc} \Sigma & \Sigma_{*}\\ \Sigma_{**}^{T} & \Sigma_{**} \end{array}\right) \end{array}\right]$$

where $\Sigma_{*}$ is the covariance of training–test data; $\Sigma_{**}$ is the covariance of the test data.

### 2.2. Bayesian Optimization: Pareto Front Approximation

To determine the lowest predicted porosity and hardness values and the matching manufacturing conditions, we used a sequential design approach with Bayesian optimization. This optimization approach lowers the costly L-PBF fabrications and seeks the global optimum in a constrained design space with the fewest possible iterations.

Using an acquisition function, sampling was directed to regions with a high likelihood of outperforming the best observation at the time. The acquisition function of DGEMO uses the expected hardness or porosity μ of the GPR posterior. In summary, the Pareto front approximation [31] is a three-part iterative approach. A stochastic sampling method was initially used to generate a set of random samples modified from the best hardness or porosity found thus far, in order to avoid local minima and achieve a balance between the exploration and exploitation of the design space regions. The second step uses a local optimization approach to arrive at a local Pareto-optimal solution for each sample. In order to cover various Pareto front areas, several optimization directions were investigated. Finally, a dense collection of solutions was obtained by extracting a first-order approximation of the Pareto front around $x_{i}$. The Jacobian and Hessian of the GP prediction were used by the Pareto front approximation algorithm to more thoroughly explore the Pareto front for optimal manufacturing conditions based on this surrogate model.

### 2.3. Bayesian Update Procedure: Batch Selection Strategy

The algorithm groups the optimal points based on their hardness or porosity values and manufacturing conditions ($x_{1}$, $x_{2}$, *…*, $x_{6}$), starting with the Pareto front approximation, to produce a number of diversity regions. The selection strategy was based on two criteria: diversity and hypervolume improvement [31]. The diversity metric combines information from both the design and performance domains, with the goal of evenly distributing the selected samples throughout the diversity regions. This approach keeps the optimization from falling into local minima and overly focusing on one high-performing area while ignoring other potentially promising regions. The selection technique tries to optimize the hypervolume improvement while requiring samples to be drawn from as many different places as possible.

The suggested approach employs an iterative integration of the Bayesian updating technique to look for the global optimal manufacturing condition. In this process, new data are gathered to update the surrogate model, and samples are created based on the predicted optimal conditions from the Bayesian optimization. The calculated optimum in an unexplored region often has a large variance, which indicates a high degree of uncertainty. As a consequence, we fabricated fresh samples in the predicted ideal fabrication configuration and collected new data in order to reduce the uncertainties related to these unexplored regions in the surrogate model. The Bayesian optimization approach was used to examine the improved surrogate model in an effort to identify the ideal manufacturing conditions for brand-new fabrications. This process was repeated until the results of the optimization converged.

## 3. Results

**Figure 3:**
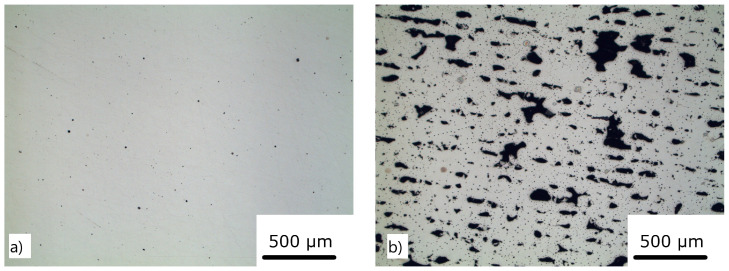
The optical image of the printed samples: (**a**) sample with energy density 144.13 J/mm${}^{3}$ with porosity 0.064%; (**b**) sample with energy density 48.23 J/mm${}^{3}$ with porosity 15.60%.

**Figure 4:**
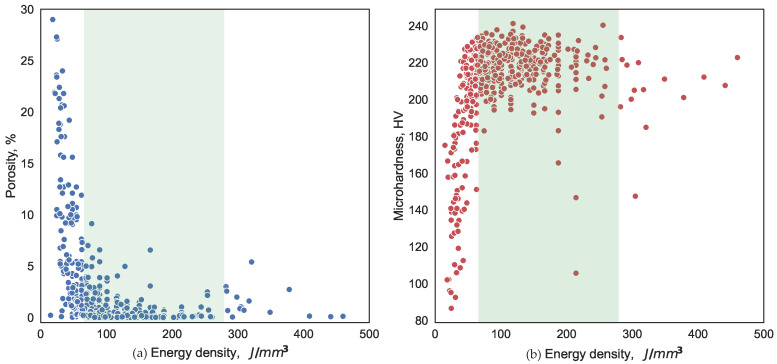
The scatter plot of (**a**) porosity (%) and (**b**) hardness (HV) as a function of VED. The shaded area represents the recommended operating window of 65–280 J/mm${}^{3}$.

After the production of the initial dataset, we could make several observations. It is evident from Figure 3a that a sufficient VED of 128.12 J/mm${}^{3}$ produced a part with porosity 0.65%. In contrast, a low VED of 48.23 J/mm${}^{3}$ (Figure 3b) resulted in a porosity of 5.27%. Figure 4 shows the target variables as a function of the VED. For a process configuration below 65 J/mm${}^{3}$, the lack of the fusion and “balling” phenomena was prevalent, which led to a high-porosity part (Figure 4a). An excessive amount of the VED burns the specimen and detrimentally affects its microstructure, leading to poor hardness (Figure 4b) and porosity. Prior to any ML model training, it was clear that the optimal process configuration was in the range between 65 and 280 J/mm${}^{3}$.

The target values obtained experimentally are shown in Table 2, with the top ten process configurations sorted by hardness in descending order and the porosity shown in Table 3 in ascending order. The obtained results concurred with previous works [16,17,36]. The operating window had a quite wide range. For instance, the laser power in Table 2 goes from 51.5 to 159 W, and the corresponding scanning speed is in the range of 335–1128 mm/s. In addition, the process configuration for the most-optimal hardness did not always coincide with the configuration with the lowest porosity. Therefore, this makes a good case for implementing MOBO.

**Figure 5:**
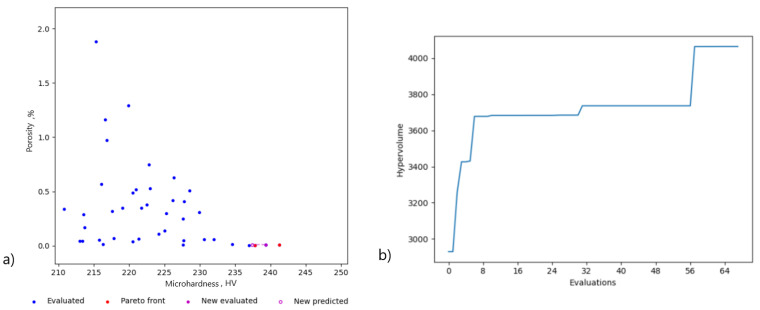
Performance results. (**a**) The scatter plot of the hardness versus porosity (Pareto front is colored red); open magenta marks represent candidate configuration, and solid magenta marks denote the suggested configuration after evaluation. (**b**) The hypervolume improvement plot on the right represents advancements toward the Pareto front.

After training the model on our subset of 57 sample points, we obtained the process configuration shown in Table 4, and the corresponding process parameters in the Pareto set are highlighted. The hypervolume indicator in Figure 5 demonstrates the actual advancements of the Pareto front over the iterations of the optimization algorithm. The proposed process configurations were experimentally validated following the same procedure discussed in Section 2. The predictions for all six parameters were almost identical, which suggests that the algorithm converged quickly.

The results in Table 5 demonstrate the actual and predicted values of the objective functions. The corresponding values of the Pareto front are highlighted. All the points fell within the confidence interval, suggesting the good predictive power of the surrogate model. The produced samples were quite dense with the actual porosity remaining below 1% and the hardness above 220 HV. Figure 5 shows the scatter plot of the two objective functions evaluated experimentally, shown in blue, and the Pareto front, colored in red, obtained after six iterations of the algorithm.

## 4. Discussion

The rate of convergence toward the Pareto front is highly dependent on the initial dataset. The closer sampled points are to the Pareto front (Figure 5a), the faster the algorithm will converge. Therefore, for the given problem, it is best to leverage previous knowledge or domain expertise to select the batch of points that is as close to the Pareto front as possible. The laser power, scanning speed, and hatch distance appear to play significant roles. It should be noted that proposed method cannot take into account feedstock-induced defects, but rather alleviates process-induced defects for the SS 316L powder.

Figure 5b represents the hypervolume improvement. It can be seen that, after just six iterations, the hypervolume indicator increased by 9% (Figure 5b) from 3700 to 4061. The specimens in the Pareto set varied in hardness from 225 to 232 HV. The highest hardness was obtained for a power of 58 W, a scanning speed of 257 mm/s, and a hatch spacing of 47 µm. The porosity of the optimal solutions spanned from 0.2 to 0.37%. The corresponding parameters for the lowest porosity are shown in Table 5.

Applying the established Bayesian framework to the optimization of the L-PBF manufactured SS 316L samples has a number of advantages. Firstly, it minimizes the number of tests required to identify the ideal manufacturing conditions. The Bayesian framework works with fewer fabrications than the complete factorial design, which tests all possible combinations of the process parameters. For instance, 15,625 tests are required for a five-level full-factorial design in six factors. If more components are included, the full-factorial design might lead to exponentially more trials. There is no need to conduct expensive tests to cover all possible combinations of the process parameters since the framework focuses on the regions that may yield the best results based on the Pareto approximation. The sustainability of our work may be a significant advantage. The approach is primarily concerned with conserving the resources used in experiments, such as time, energy, and materials.

Secondly, the process design consideration could be improved. For instance, if we would like to increase the build rate, we might add another objective function that searches for high-quality components that take the least amount of time to build. However, in order to produce AM components quickly, the scanning speed might be increased. However, this will result in molten pool elongation, and depending on the scanning speed, the liquid pool may become unstable owing to the fragmentation of the single molten pool into discrete puddles of liquid, causing a discontinuity in the geometry. Increasing the layer thickness is another way to shorten the build time. However, this will affect the surface roughness and cause additional post-processing steps.

The present work can be further extended by applying the MOBO algorithm to other printed material characteristics, features such as tensile properties, fatigue resistance [37], impact load resistance, etc. Exploring different model hyperparameters could also reveal new process configurations. Augmenting the data-driven approach with a mechanistic model could enhance predictions and make them less susceptible to the quality of the data. Incorporating dimensionless numbers and geometry information can further improve the predictive power of the model. In this work, we looked at GP-based models, but other ML models such as boosted trees and neural networks could be employed [38,39] as well. Finally, automating tasks by developing high-throughput automated experimentation [20] for the L-PBF process can speed up the research efforts even more.

## 5. Conclusions

In this study, a novel MOBO algorithm was applied for the optimization of the L-PBF process. The present work developed an optimal processing window that produced SS 316L effectively and affordably. The research findings can be summarized as follows:

- The model trained on relatively small batch of data quickly found three points on the Pareto front in just six iterations.
- The highest value of the hardness obtained empirically was 241.3 HV, corresponding to a VED of 118.5 J/mm${}^{3}$, with a power of 133 W, a scanning speed of 850 mm/s, and hatch spacing of 66 µm.
- The highest relative density part had a porosity of 0.0007% and the following parameters: VED 119.72 J/mm${}^{3}$, power 108 W, scanning speed 465 mm/s, hatch spacing 97 µm.
- The VED that was explored by the algorithm lied in the range of 240–265 J/mm${}^{3}$; the hardness of the produced parts was 224–235 HV, and the porosity was in the range of 0.2–0.37%.
- The recommended processing window corresponded to the parts manufactured with an energy density that lied in the range of 65–280 J/mm${}^{3}$.
- The trained model prescribed the following parameters to ensure quality parts: 58 W, 257 mm/s, 45 µm, with a scan rotation angle of 131 degrees.

## Figures and Tables

**Table 1:** Process parameter range.

Parameters	Minimum Value	Maximum Value
Time (min)	1	4
Gas circulation speed (m/s)	1.5	4
Laser power (W)	30	175
Scan speed (mm/s)	100	3000
Hatch distance (µm)	40	120
Scan angle (degrees)	0	150

**Table 2:** Empirically obtained process parameter configurations sorted by highest hardness.

Time, min	Gas Feed, m/s	Power, W	Speed, mm/s	Hatch Spacing, µm	Energy Density, J/mm${}^{3}$	Angle, °	Hardness, HV
2	3.5	133.0	850.0	66.0	118.5	44.0	241.3
2	3.5	154.0	335.0	90.0	255.4	48.0	240.3
2	3.5	123.0	583.0	79.0	133.5	18.0	239.3
2	3.5	159.0	1128.0	75.0	93.9	50.0	237.8
2	4.0	74.3	448.6	70.0	118.3	74.9	237.0
3	2.5	113.0	910.0	80.0	77.6	150.0	236.0
2	3.5	101.0	505.0	87.0	114.9	5.0	235.4
3	3.5	51.5	726.9	41.0	86.4	10.7	235.1
3	2.5	147.0	490.0	90.0	166.7	150.0	235.0
2	3.5	114.0	454.0	67.0	187.4	41.0	235.0

**Table 3:** Empirically obtained process parameter configurations sorted by lowest porosity.

Time, min	Gas Feed, m/s	Power, W	Speed, mm/s	Hatch Spacing, µm	Energy Density, J/mm${}^{3}$	Angle, °	Porosity, %
2	3.5	108.0	465.0	97.0	119.72	77.0	0.0007
2	3.5	163.0	616.0	71.0	186.35	51.0	0.0013
2	3.5	131.0	955.0	77.0	89.07	83.0	0.0042
2	3.5	101.0	505.0	87.0	114.94	5.0	0.0043
2	3.5	159.0	1128.0	75.0	93.97	50.0	0.0058
2	3.5	146.0	606.0	88.0	136.89	8.0	0.0067
2	3.5	137.0	628.0	82.0	133.02	25.0	0.0071
2	3.5	138.0	914.0	92.0	82.06	30.0	0.0074
2	3.5	133.0	850.0	66.0	118.54	44.0	0.0075
2	3.5	163.0	1268.0	74.0	86.86	35.0	0.0083

**Table 4:** Process parameters suggested by the DGEMO algorithm. Highlighted rows indicate the Pareto set.

Time, min	Gas Feed, m/s	Power, W	Speed, mm/s	Hatch Spacing, µm	Energy Density, J/mm${}^{3}$	Angle, °
3	3.5	59.0	254.0	45.0	258.1	123.0
3	3.5	58.0	257.0	47.0	240.1	131.0
3	3.5	60.0	257.0	44.0	265.3	123.0
2	3.5	59.0	256.0	45.0	256.1	123.0
2	3.5	59.0	258.0	45.0	254.1	124.0
2	3.5	59.0	259.0	44.0	258.9	123.0

**Table 5:** Predicted versus actual target variables. Highlighted rows indicate the Pareto front.

Predicted Hardness (Std.), HV	Actual Hardness, HV	Predicted Porosity (Std.), %	Actual Porosity. %
248.0 ± 18.0	231.0	0.510 ± 2.918	0.200
248.0 ± 17.7	235.0	0.510 ± 2.854	0.310
248.0 ± 18.5	225.0	0.490 ± 2.980	0.370
248.0 ± 17.9	229.0	0.520 ± 2.916	0.260
248.0 ± 18.4	224.0	0.480 ± 2.930	0.200
249.0 ± 18.6	232.0	0.450 ± 2.948	0.210

## Data Availability

The data presented in this study are available on request from the corresponding author.

## Abbreviations

The following abbreviations are used in this manuscript:

AM	Additive manufacturing
L-PBF	Laser powder bed fusion
MOBO	Multi-objective Bayesian optimization
ML	Machine learning
GP	Gaussian process
